# Role of gut microbiota dysbiosis in the pathogenesis of non-alcoholic fatty liver disease among obese individuals

**DOI:** 10.6026/973206300212868

**Published:** 2025-08-31

**Authors:** Vijaya Krishna Prasad Vudathaneni, Swetha Bharathi Nadella, Raja Mogallapu, Kalikrishna Varaprasad Movva, Ramanarayana Boyapati

**Affiliations:** 1 Department of Internal Medicine, Columbus Regional Hospital, Columbus, Indiana, United States of America; 2 Department of Behavioral Medicine and Psychiatry, West Virginia University School of Medicine, West Virginia University, Morgantown, United States of America; 3 Department of Anaesthesiology, Anaesthetics, Principal House Officer, Mackay Base Hospital, Mackay, Queensland, Australia; 4 Department of Periodontology, Sibar Institute of Dental Sciences, Takkellapadu, Guntur andhra Pradesh, India

**Keywords:** Gut microbiota, dysbiosis, non-alcoholic fatty liver disease (NAFLD), obesity, hepatic steatosis, liver inflammation, microbial diversity, 16S rRNA sequencing

## Abstract

Non-alcoholic fatty liver disease (NAFLD) is highly prevalent in obese individuals, and gut microbiota dysbiosis is increasingly recognized as a major contributor to its pathogenesis. In this cross-sectional study of 80 obese participants (40 NAFLD and 40 controls), 16S rRNA sequencing revealed significantly reduced microbial diversity in NAFLD patients (Shannon index 2.9 vs. 3.6; p=0.001), with increased Firmicutes, *Enterobacteriaceae*, and Ruminococcus, and decreased *Bacteroidetes*. These patients also showed elevated liver enzymes (ALT, AST) and inflammatory markers (TNF-α, IL-6). A strong correlation was observed between gut dysbiosis and hepatic steatosis (r=0.72; p<0.001), highlighting the potential of microbiota modulation as a therapeutic strategy for NAFLD.

## Background:

Non-alcoholic fatty liver disease (NAFLD) represents a spectrum of hepatic disorders ranging from simple steatosis to non-alcoholic steatohepatitis (NASH), fibrosis and potentially cirrhosis, occurring in the absence of significant alcohol consumption. With the global rise in obesity and metabolic syndrome, NAFLD has emerged as the most common chronic liver disease, affecting approximately 25% of the global population and up to 80% of obese individuals [1, 2]. The complex interplay between metabolic dysfunction, insulin resistance, inflammation and genetic predisposition underlies the pathophysiology of NAFLD. Recent evidence has highlighted the pivotal role of the gut-liver axis in NAFLD development. The gut microbiota, comprising trillions of microorganisms, is essential for maintaining intestinal homeostasis, energy metabolism and immune regulation [3]. Dysbiosis, or the disruption of the normal gut microbiota composition, has been implicated in metabolic disorders, including obesity, type 2 diabetes and NAFLD [4]. Mechanistically, gut dysbiosis may influence hepatic fat accumulation through increased intestinal permeability, endotoxemia, altered short-chain fatty acid production and modulation of bile acid metabolism [5, 6]. Several studies have demonstrated that individuals with NAFLD exhibit reduced microbial diversity and an imbalance in specific bacterial phyla, particularly a higher Firmicutes-to-*Bacteroidetes* ratio, which may contribute to metabolic endotoxemia and hepatic inflammation [7, 8]. Furthermore, overgrowth of pathogenic bacteria such as *Enterobacteriaceae* and underrepresentation of beneficial genera like *Bifidobacteria* and *Lactobacillus* have been observed in NAFLD patients [9]. These microbial alterations are believed to facilitate lipogenesis and impair lipid oxidation within the liver, promoting steatosis and inflammation [10]. Understanding the association between gut microbiota dysbiosis and NAFLD is critical, especially in the context of obesity, where both conditions frequently coexist. Therefore, it is of interest to investigate the composition of gut microbiota in obese individuals with and without NAFLD and to assess the relationship between microbial alterations and hepatic steatosis severity.

## Materials and Methods:

## Study design and population:

A cross-sectional observational study was conducted over a 6-month period at a tertiary care center. A total of 80 obese participants (BMI ≥30 kg/m^2^), aged between 25 and 60 years, were recruited. Participants were divided into two groups: Group A consisted of 40 obese individuals diagnosed with non-alcoholic fatty liver disease (NAFLD) and Group B included 40 age- and sex-matched obese controls without NAFLD. NAFLD diagnosis was confirmed using hepatic ultrasonography and transient elastography (FibroScan), in the absence of significant alcohol intake or other chronic liver diseases.

## Inclusion and exclusion criteria:

Subjects with a history of alcohol consumption exceeding 20 g/day for men and 10 g/day for women, viral hepatitis, autoimmune liver disease, or those on medications affecting liver function or gut microbiota (e.g., antibiotics, probiotics within 3 months) were excluded.

## Anthropometric and biochemical assessment:

Height and weight were measured to calculate body mass index (BMI). Waist circumference and blood pressure were also recorded. Fasting blood samples were obtained to evaluate liver function tests (ALT, AST), lipid profile, fasting glucose, insulin and inflammatory markers such as TNF-α and IL-6 using enzyme-linked immunosorbent assay (ELISA) kits. Homeostasis Model Assessment for Insulin Resistance (HOMA-IR) was calculated to assess insulin resistance.

## Gut microbiota analysis:

Stool samples were collected in sterile containers and stored at -80°C until analysis. Microbial DNA was extracted using a standardized commercial kit. The V3-V4 regions of the bacterial 16S rRNA gene were amplified and sequenced using an Illumina MiSeq platform. Bioinformatic processing of the sequences was performed using QIIME 2 pipeline, including quality filtering, taxonomic assignment and alpha/beta diversity estimation.

## Statistical analysis:

Data analysis was carried out using SPSS software version 25.0 (IBM Corp., Armonk, NY, USA). Continuous variables were presented as mean ± standard deviation, while categorical variables were expressed as percentages. Differences between groups were assessed using Student's t-test or Mann-Whitney U test for continuous data and chi-square test for categorical variables. Correlation between gut dysbiosis and hepatic steatosis was evaluated using Pearson or Spearman correlation coefficients. A p-value of less than 0.05 was considered statistically significant.

## Results:

A total of 80 obese participants were included in the study, with 40 individuals diagnosed with non-alcoholic fatty liver disease (Group A) and 40 obese controls without NAFLD (Group B). The demographic and clinical characteristics of both groups are presented in Table 1 (see PDF). No statistically significant differences were observed in mean age or sex distribution between the two groups (p>0.05). However, individuals with NAFLD had significantly higher BMI, waist circumference and HOMA-IR scores (p<0.05). Group A showed significantly elevated liver enzymes compared to Group B, with mean ALT and AST values of 68 ± 14 U/L and 56 ± 11 U/L, respectively, versus 35 ± 10 U/L and 28 ± 8 U/L in controls (p<0.001). Additionally, inflammatory markers such as TNF-α and IL-6 were markedly higher in the NAFLD group (Table 2 - see PDF). Microbiota analysis revealed a notable reduction in alpha diversity among NAFLD patients, with a lower Shannon diversity index (2.9 ± 0.4) compared to controls (3.6 ± 0.5, p=0.001). Taxonomic composition analysis indicated a higher Firmicutes/*Bacteroidetes* ratio in the NAFLD group. Pathogenic genera such as *Enterobacteriaceae* and Ruminococcus were significantly more abundant in Group A, while beneficial microbes like Bifidobacterium were reduced (Table 3 - see PDF). Correlation analysis demonstrated a strong positive association between the dysbiosis score and hepatic fat content measured by FibroScan (r=0.72, p<0.001), suggesting that worsening microbial imbalance is linked to increasing severity of hepatic steatosis. These findings collectively indicate that obese individuals with NAFLD exhibit significant microbial alterations, elevated inflammatory markers and metabolic dysfunction, reinforcing the potential role of gut dysbiosis in disease pathogenesis.

## Discussion:

The current study provides evidence supporting a significant association between gut microbiota dysbiosis and the development of non-alcoholic fatty liver disease (NAFLD) in obese individuals. The findings align with previous literature indicating that altered gut microbial composition may play a contributory role in NAFLD pathogenesis by influencing host metabolism, immune responses and intestinal barrier integrity [1, 2]. Obese individuals with NAFLD in our study exhibited a markedly reduced microbial diversity and a disrupted Firmicutes-to-*Bacteroidetes* ratio. This shift has been consistently observed in both human and animal studies and is often interpreted as a microbial signature of metabolic disorders such as obesity and NAFLD [3, 4]. The increased relative abundance of *Firmicutes* may promote energy harvesting from indigestible polysaccharides, enhancing lipogenesis and hepatic fat accumulation [5]. Simultaneously, the depletion of *Bacteroidetes*, known for their anti-inflammatory and metabolic regulatory roles, may exacerbate the inflammatory state characteristic of steatohepatitis [6]. In line with earlier findings, this study also documented elevated levels of pro-inflammatory cytokines TNF-α and IL-6 in NAFLD patients [7]. These cytokines are key mediators of hepatic inflammation and have been implicated in the progression from simple steatosis to steatohepatitis and fibrosis [8]. The translocation of bacterial endotoxins, facilitated by increased gut permeability due to dysbiosis, may activate Kupffer cells and trigger an immune cascade that intensifies hepatic injury [9, 10]. Additionally, overrepresentation of *Enterobacteriaceae* and Ruminococcus in the NAFLD group is noteworthy. Members of the *Enterobacteriaceae* family are capable of producing endotoxins such as lipopolysaccharide (LPS), which can induce systemic low-grade inflammation and insulin resistance, further fueling liver damage [11]. On the other hand, beneficial genera such as Bifidobacterium and *Lactobacillus*, which have protective roles against gut barrier dysfunction and inflammation, were significantly reduced [12, 13]. This inverse relationship between beneficial and pathogenic microbes underscores the importance of microbial balance in maintaining metabolic homeostasis. Our observation of a strong positive correlation between dysbiosis scores and hepatic steatosis severity supports the hypothesis that the gut-liver axis plays a pivotal role in NAFLD development. Emerging studies have shown that microbial metabolites such as short-chain fatty acids (SCFAs), secondary bile acids and ethanol can modulate liver metabolism and inflammation [14]. In dysbiotic states, these metabolites are produced in abnormal proportions, contributing to hepatic lipotoxicity and immune dysregulation [15]. The use of 16S rRNA gene sequencing in this study allowed for precise profiling of microbial taxa, supporting recent trends in microbiome research that link specific genera with metabolic disease phenotypes [16]. However, it is important to recognize that while the association is evident, causality cannot be definitively established due to the cross-sectional nature of this study. Longitudinal studies and controlled clinical trials are needed to better understand the temporal relationship between microbial shifts and liver pathology. Furthermore, dietary factors, antibiotic use and genetic polymorphisms influencing host-microbiome interactions may serve as confounding variables that were not fully accounted for. Nonetheless, the consistency of our findings with previously published work lends credibility to the emerging concept of targeting the gut microbiota as a potential therapeutic strategy in NAFLD management [17, 18]. Probiotic, prebiotic and synbiotic interventions, along with dietary modifications and fecal microbiota transplantation, are currently being explored for their efficacy in restoring microbial equilibrium and ameliorating hepatic steatosis [19, 20]. Nonalcoholic fatty liver disease (NAFLD) is a hepatic manifestation of metabolic syndrome and is closely linked to the rising global prevalence of obesity, insulin resistance, and type 2 diabetes mellitus. Its pathogenesis is multifactorial, involving genetic predisposition, dietary patterns, lifestyle factors, and increasingly, the gastrointestinal microbiota [21]. Gut dysbiosis has been proposed to influence NAFLD progression through mechanisms such as increased intestinal permeability, bacterial translocation, and production of endotoxins like lipopolysaccharides, which trigger systemic inflammation and hepatic injury [22]. Furthermore, gut microbiota (GM) dysbiosis and microbial metabolites play a crucial role in the development and pathogenicity of NAFLD [23]. The pathogenesis of NAFLD is complex and multifactorial, involving environmental, genetic and metabolic factors [24, 25]. Hence, further research is warranted to clarify how gut microbiota interact with metabolic risk factors and whether microbiota-targeted therapies, such as probiotics, prebiotics, or fecal microbiota transplantation, could provide effective interventions in NAFLD management.

## Conclusion:

Growing body of evidence implicating gut microbiota dysbiosis in the pathogenesis of NAFLD among obese individuals is shown. Thus, we show the potential of microbiota-based diagnostics and therapeutics in the personalized management of metabolic liver diseases.

## References

[1] Safari Z (2019). Cell Mol Life Sci..

[2] Chen HT (2020). World J Gastroenterol..

[3] Cho MS (2018). J Microbiol..

[4] Hu H (2020). J Gastroenterol..

[5] Xie C (2019). Nutrients..

[6] Suk KT, Kim DJ. (2019). Expert Rev Gastroenterol Hepatol..

[7] Jayachandran M (2023). Rev Endocr Metab Disord..

[8] Chen J (2020). Int J Mol Sci..

[9] Vallianou N (2021). Biomolecules..

[10] Leung C (2016). Nat Rev Gastroenterol Hepatol..

[11] Kuraji R (2023). World J Gastroenterol..

[12] Khan A (2021). Int J Biol Sci..

[13] Leylabadlo HE (2020). Eur J Clin Microbiol Infect Dis..

[14] Fernández-Musoles R (2020). Nutr Hosp..

[15] Nagashimada M (2021). Int J Mol Sci..

[16] Mijangos-Trejo A (2023). Int J Mol Sci..

[17] Ma J (2017). Nutrients..

[18] Kirpich IA (2015). Clin Biochem..

[19] Koopman N (2019). Aliment Pharmacol Ther..

[20] Park JW (2021). Int J Mol Sci..

[21] Jasirwan CM (2019). Biosci Microbiota Food Health..

[22] Guo L (2021). Surg Pract Sci..

[23] Yaghmaei H (2024). Obes Med..

[24] Jennison E (2021). Clin Mol Hepatol..

[25] Shen F (2017). Hepatobiliary Pancreat Dis Int..

